# Numerical Evaluation of the Hygrothermal Performance of a Capillary Active Internal Wall Insulation System under Different Internal Conditions

**DOI:** 10.3390/ma15051862

**Published:** 2022-03-02

**Authors:** Dobrosława Kaczorek

**Affiliations:** Thermal Physics, Acoustics and Environment Department, Building Research Institute (ITB), Filtrowa 1 Str., 00-611 Warsaw, Poland; d.kaczorek@itb.pl

**Keywords:** hygrothermal simulation, capillary active internal insulation, mould risk, moisture effects

## Abstract

In certain situations, internal insulation is the only possible renovation option. However, it is risky where there is high humidity in a building and ventilation is not working sufficiently. The internal insulation retrofit changes the original thermal and moisture balance of a wall, therefore, it is necessary to carefully design it already at the initial stage. This paper analyses four interior insulation systems based on open diffusion capillary active materials: wood fibreboards (flex and rigid), perlite boards, and microporous calcium silicate. The hygrothermal performance under the climatic conditions of Central Europe (Poland) was assessed using the WUFI Plus software, taking into account the dynamic variation of indoor and outdoor conditions. The analysis included three insulation thicknesses with different ventilation rates and varying moisture loads. The results show that the hygrothermal properties of the wall change with the increase of insulation thickness and depend on the individual moisture properties of the material. In addition, both the reduction of moisture load and more intensive air exchange improve the hygrothermal properties at the interface between the insulation and the wall. Of all the solutions analysed, the system with perlite board and the system with wood fibreboard showed the worst hygric properties. Conversely, the highest risk of mould and interstitial condensation was recorded for the flex wood fibreboard solution.

## 1. Introduction

As the construction sector is responsible for approx. 40% of Europe’s greenhouse gas emissions [1], the European Commission has begun to take an interest in the industry’s activities. In order to reach the ambitious European climate targets for reducing greenhouse gas emissions, The Energy Performance of Buildings Directive (EPBD) [2] highlights the need for member states to develop long-term strategies for building renovation. As a result, in January 2021, new requirements for the thermal protection of buildings came into force in Poland, according to which the heat transfer coefficient for external walls must be less than U = 0.2 W/(m^2^·K), both for newly constructed buildings and buildings undergoing thermal efficiency improvement. Not all buildings, however, are suitable for insulation being fitted on the outside of walls. In such a case, internal insulation may be the only feasible solution [3], although it is often considered more risky due to the presence of thermal bridges [4,5,6] and the increased likelihood of interstitial condensation [7,8,9,10]. In addition, this type of solution increases the diffusion resistance between the existing wall and the occupied zone, which reduces the potential for the wall to dry inwards [9,11]. In turn, higher moisture levels can lead to mould growth on the internal surface [9,12,13,14] or damage to the timber elements of the wall [9,13,15]. In order to solve the issues concerning moisture accumulation inside the wall, and to improve its thermal properties, new solutions are constantly searched for. An example could be the thermal paint with a ceramic material employed as a special filler. Despite the fact that the ceramic additive works well in other solutions [16], unfortunately, it does not improve the thermal properties of this paint, nor the thermal insulation of the building structure on which it was applied [17]. Another option, increasingly popular in recent years, are capillary active systems with open diffusion [18]. This group of materials includes, among others, autoclaved aerated concrete (AAC) and calcium silicate (Ca-Si). Some studies [12,19,20] have shown that the use of capillary active insulation materials based on calcium silicate (Ca-Si) significantly reduces the moisture content in walls. The results, however, may have been influenced by the application of additional wind-driven rain (WDR) protection by coating the external layer with a hydrophobic agent or low internal moisture load. Other studies [21,22] have shown high relative humidity (RH) levels at the interface between the wall and insulation, depending on various factors such as wall thickness, rain load, or the hygroscopicity of materials.

The results of many previous simulation studies [12,15,22,23,24] on the importance of insulation thickness prove that increased thickness increases the risk of moisture problems. In contrast, Nielsen et al. [25] and Straube et al. [9] suggested that insulation thickness is only of minor importance and that the condensation risk associated with outward diffusion is a minor problem compared to the amount of moisture introduced into the wall due to WDR. Conversely, studies [3,22,26] indicated that critical relative humidity (RH) levels decreased as masonry thickness increased. However, little information can be found in the literature regarding the influence of mortar properties on the performance of internal insulation, while the properties of the mortars themselves are the subject of numerous works [27,28].

Another frequently used measure to improve hygrothermal conditions at the interface between the wall and the insulation is to reduce internal moisture loads in the rooms, especially during the heating season. Both field [11,29] and simulation studies [8,18,22,30,31] indicated the improvement of hygrothermal performance at critical locations resulting from indoor moisture load reduction.

The group of capillary active materials also includes biomaterials, e.g., insulation based on wood fibreboards. This type of insulation is most often used to fill finished structures [32,33]; however, appropriately designed insulation can also be used as internal insulation. An assessment of the suitability of this type of insulation for internal use in the Central European region is presented in the work by Kočí et al. [34]. Furthermore, Wegerer et al. [35] evaluated different wood fibre interior insulation systems based on the results of measurements performed on two demonstration objects.

In light of the above, the aim of the conducted research was the evaluation of the hygrothermal behaviour of four internal insulation systems in the climatic conditions of the city of Kraków, Poland (temperate climate zone, middle latitude). The Glaser model [36,37] has been used most commonly to assess the hygrothermal processes in external walls. This model, however, is not suitable for the assessment of walls containing hygroscopic materials in which variable moisture processes occur simultaneously in multiple phases [38]. Furthermore, in many studies on heat and mass transfer issues in the building envelope, the internal environmental conditions were assumed to be constant [39] or determined in accordance with EN 15026 [40], where indoor air temperature and relative humidity depend linearly on the outdoor temperature [41,42]. There are many studies on the thermal comfort and energy conservation of buildings, where dynamic thermal comfort is increasingly considered in the thermal environment of buildings [43,44,45]. There are, however, relatively few studies on heat and moisture transfer in walls in dynamic indoor thermal environments using 3D models. Therefore, in this study, research based on the Heat and Mass Transfer (HAMT) simulation model using the WUFI Plus simulation tool [45,46], for different solutions of internal conditions was carried out.

This research analyses four interior insulation systems based on open diffusion capillary active materials: wood fibreboards (flex and rigid), perlite boards, and microporous calcium silicate. The research focused primarily on the analysis of the hygrothermal performance of external walls under varying indoor environmental conditions in the room, taking into account different ventilation solutions and occupancy schedules. The choice of variants for the analyses was aimed at reflecting the typical scenarios of using the typical room in the residential building. The analyses carried out, made it possible to identify the least favourable solution. The energy and economic analyses have been presented in an earlier article [47].

## 2. Materials and Methods

### 2.1. Simulation Method

In order to test the hygrothermal performance of individual cases, numerical simulations using the WUFI Plus software (version 3.1.0.3) were carried out. For these simulations, from which information on the hygrothermal behaviour of the external walls and the internal hygrothermal conditions was obtained, various comparative conditions were established. Concerning the wall, total moisture content, temperature, and RH on its internal surface and in the critical cross-section at the interface between the additional insulation and the wall were analysed.

A hypothetical room model was built; it was assumed that this room is an internal room and that neighbouring rooms are of the same type, thus excluding heat and mass through internal walls. It was also assumed that the examined room with the following dimensions: length—4.0 m, width—5.5 m, height—2.8 m, is a family room with a 2.9 m^2^ window facing south.

Tests have been conducted on a base wall made of two layers: 25 cm thick solid brick and 11 cm thick extruded polystyrene (EPS) external insulation with a heat transfer coefficient of 0.23 W/(m^2^·K), insulated from the inside using various internal insulation systems so that the entire wall meets the thermal protection requirements effective in Poland since 2021 (U ≤ 0.2 W/(m^2^·K)). The configuration of individual solutions together with material data is presented in Table 1. The analyses were carried out for three thicknesses of the insulation material: 4 or 5 cm (depending on the type), 8 and 12 cm. The values of the physical properties of the insulation materials adopted for calculations were determined as part of the H-house project in the Building Research Institute’s laboratory [48]. The properties of other materials were adopted based on the WUFI Plus database.

The walls were modelled as the walls in a tall building (>20 m) since higher buildings are most exposed to the effect of driving rain than lower ones. In the absence of an impregnation layer on the exterior surface of the wall, driving rain water absorption coefficient of up to 70%, depending on the slope and type of wall, was adopted. The computations were carried out over a period of 3 years. Hygrothermal computations started in January with internal moisture content corresponding to 80% relative humidity and a temperature of 20 °C.

### 2.2. External Conditions

The exterior conditions were derived from the WUFI Plus database for the city of Kraków, Poland. The weather data included hourly values of temperature, relative humidity, solar radiation, barometric pressure, long-wave counter radiation, and rain load. Kraków is situated at 50.03° north latitude and 19.56° east longitude, in a temperate climate zone (middle latitude). The average temperature in Kraków is: annual, 8.28 °C, in August, 17.5 °C, in January, −1.3 °C.

### 2.3. Internal Conditions

The tests analysed four different scenarios with respect to ventilation rates (Table 2). In all analysed variants, the minimum temperature for the heating season was assumed at the level of 20 °C. In variants V1, V2, and V3 cooling was assumed in the summer season (max. temp. 26 °C). Variant V1 assumes that the room is ventilated by infiltration only, with relatively tight windows, assuming an hourly air exchange value of n = 0.2 h^−1^. In variant V2, the air exchange rate was increased to n = 0.5 h^−1^. In variant V3, infiltration was increased by assuming that the room is ventilated twice a day (ajar window over a period of one hour at 8 a.m. and 6 p.m., n = 4.0 h^−1^). In contrast, variant V4 analysed the same conditions as variant V3 but without cooling.

Furthermore, it was assumed that two adults and one child would use the room. In order to simulate the impact of moisture loads, two different variants of occupancy schedule were adopted:

- constant from 8 a.m. to 10 p.m. for weekdays and weekends, assuming that the room is used all the time during this time
- with variable occupancy schedule for weekdays and weekends according to Table 3.

## 3. Moisture Condition Assessment

According to the mechanisms of moisture transport through the building envelope, water vapour diffusion typically occurs from a high-temperature environment to a cooler environment, as does liquid transport from the high relative humidity side to the low relative humidity side. This is, however, dependent on the properties of the individual materials and the construction of the envelope; in some cases, there may be a situation where moisture will accumulate in places where the transport of moisture is limited [34]. During the coldest and warmest seasons, when outdoor relative humidity is high, moisture transport through the envelope is intensified by larger temperature and relative humidity gradients, and moisture accumulation within the envelope can increase. The combined effects of temperature and moisture cause the deterioration of building materials, especially when moderate or high temperatures are combined with high humidity for extended periods of time. In 2002, Beaulieu, Bomberg et al. [49] defined the Relative Humidity and Temperature (RHT) index as an index used to quantify the hygrothermal condition of an envelope, illustrating the duration of coexisting moisture and thermal conditions above a pair of threshold levels. In 2005, Mukhopadhyaya et al. [50] developed the Relative Humidity and Temperature Time (RHTT) index, defined below to indicate favourable conditions for initiating the onset of moisture damage, including both the RHT index and the calculated time of wetness (TOW). Higher RHTT values indicate an increased likelihood of envelope damage. The RHTT index, determined from Equations (1)–(3) below, was used to evaluate conditions conducive to potential mould growth in the envelope [51]:

(1) $${\mathrm{RH}}_{\mathrm{potential}}=\left\{\begin{array}{c} \mathrm{RH}-{\mathrm{RH}}_{\mathrm{critical},}\;\mathrm{if}\;\mathrm{RH}>{\mathrm{RH}}_{\mathrm{critical}}\\ 0,\;\mathrm{if}\;\mathrm{RH}\leq {\mathrm{RH}}_{\mathrm{critical}} \end{array}\right.$$

(2) $$T_{\mathrm{potential}}=\left\{\begin{array}{c} T-T_{\mathrm{critical},}\;\mathrm{if}\;T>T_{\mathrm{critical}}\\ 0,\;\mathrm{if}\;T\leq T_{\mathrm{critical}} \end{array}\right.$$

(3) $$\mathrm{RHTT}=\mathrm{TOW}\cdot \overset{\mathrm{total}\;\mathrm{hours}}{\underset{1}{\sum }}{\mathrm{RH}}_{\mathrm{potential}}\cdot T_{\mathrm{potential}}$$

where RH_potential_, the moisture potential for moisture damage (%); T_potential_, the temperature potential for moisture damage (°C); RH_critical_, the critical relative humidity level above which moisture damage is more likely to occur (%); T_critical_, the critical temperature level above which moisture damage is more likely to occur (°C); TOW, the calculated time of wetness (h) when RH > 80% and T > T_critical_.

The value of critical relative humidity and temperature depends on the interaction between materials, surfaces, the surrounding environment, and moisture damage. Referring to previous studies on the possibility of mould growth using the RHTT index [52], the following values were adopted in tests: relative humidity (RH_critical_) 80%, temperature (T_critical_) 5 °C.

## 4. Results and Discussion

### 4.1. Total Water Content

The basic parameter used to assess the hygric properties of a wall is the total moisture content in it. The course of the total moisture content in the wall during successive years of building exploitation shows whether the wall dries out or accumulates moisture. Frequently, moisture content that increases in time indicates numerous design errors, which may lead to the occurrence of mould on the internal surface, damage to the wall as a result of water freezing, as well as to the increase of heat losses.

**Figure 1:**
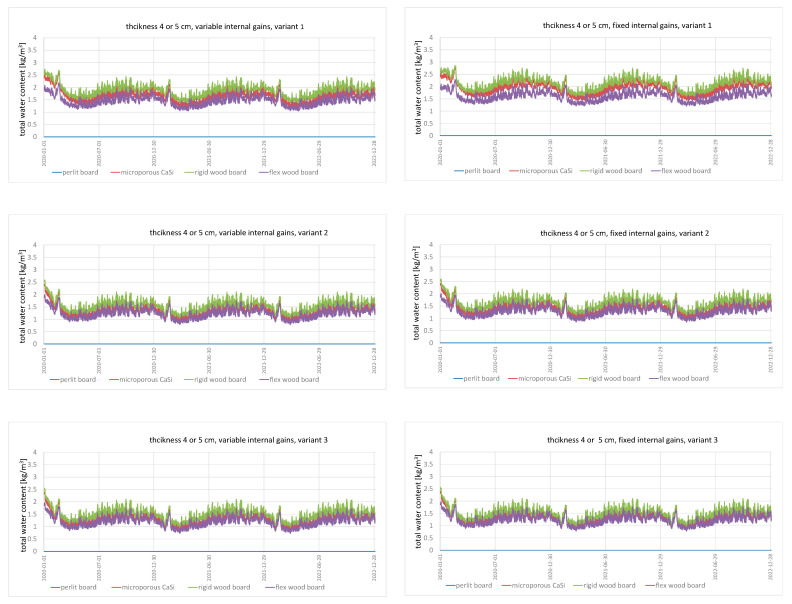

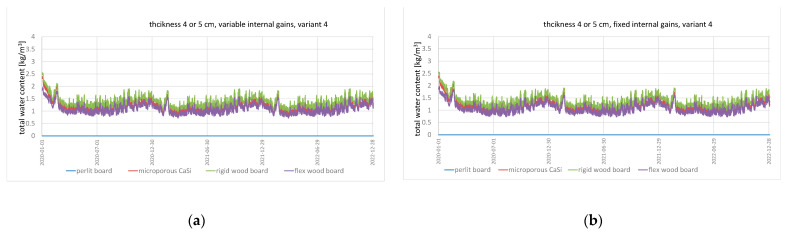
Variations in total water content in walls for 4 or 5 cm internal insulation thickness: (**a**) variable internal gains; (**b**) fixed internal gains.

Analyses of total moisture content in the wall for individual variants were performed. Figure 1 only presents selected results for 4 or 5 cm insulation thickness for the variable and constant occupancy schedule, respectively.

The wall assemblies analysed in all variants show annual cyclic fluctuations of moisture content caused by seasonality. Similar results were found in Ref. [53]. In the initial period of the simulation, in January, one can see a decrease in the walls’ moisture content, which indicates that the walls are drying out. Then, after reaching the minimum level, the total moisture content in the walls start to increase, reaching the maximum level in the summer. This is due to the fact that the winter months (December, January) are the months with the least amount of rainfall, while the summer months (June–August) are characterised by peak rainfall. The obtained results are consistent with the previous studies [52]. In the case of variants V1, V2 and V3, all walls reach the minimum level of moisture in April and the maximum level in September. These levels differ for variant V4, with a minimum level in July and a maximum level in December. This is due to the higher internal temperature that results from the lack of cooling in the summer. The higher temperature translates into higher content of water vapour in the air, which in turn causes walls made of capillary-active diffusion open insulation materials to absorb more moisture from the air.

Table 4 presents a summary of the maximum and minimum values of total moisture content for all analysed cases.

When analysing max. and min. values of the total moisture content in a wall, one might observe repeatability. For wall A (perlite board) and for wall C (rigid wood fibreboard), both in the variant with constant and variable occupancy schedules, the same values were obtained. Additionally, the maximum values for variants V3 and V4, apart from having the same results for walls A and C, reached the same value in both constant and variable occupancy schedules. In the case of 4 or 5 cm thick insulation, the highest value of total moisture content was achieved by wall A (for all ventilation variants). For other thicknesses, 8 and 12 cm, such value was achieved by variants with more intensive ventilation: V2, V3, V4, wall C. The lowest total moisture content was achieved by wall D (flex wood fibreboard), irrespective of the insulation thickness and ventilation variant. This is largely due to the sorption properties of capillary open materials, which were compared, among others, by Zhao et al. [8] and Koči et al. [34] and the value of the diffusion resistance of internal finishes. According to the research carried out by Hansen [23], the combination of these two properties may significantly reduce drying out into a room and increase the moisture content of a partition. This is the case for partition A. The perlite board is characterised by the highest sorption, and the mineral plaster layer has the highest value of diffusion resistance of all the cases considered.

In addition, one might see that the total moisture content of the wall increases with the increase of insulation thickness. Many researchers have also studied the effect of insulation thickness on the hygric behaviour of the wall [12,23,53]. Their research confirms that the ability of the wall to accumulate moisture increases with the increase of insulation thickness. In addition, one might spot that higher insulation thicknesses result in greater differences between the materials, especially for variant V1, which has a low air exchange rate.

### 4.2. Temperature and RH at Interior Surface

**Figure 2:**
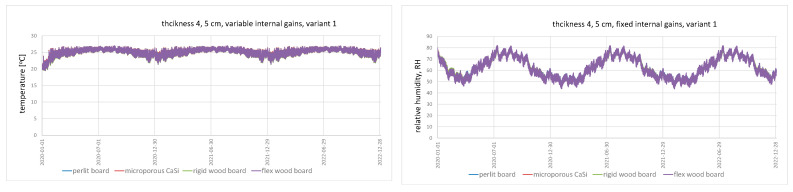

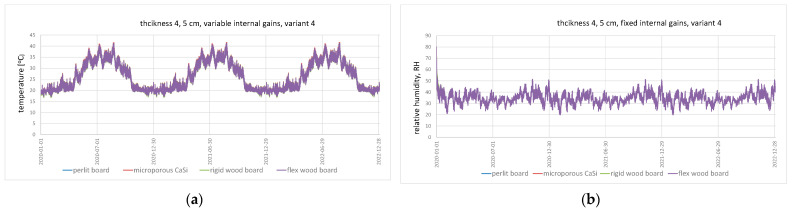
Annual seasonal fluctuations in temperature and relative humidity on the inner surface for 4 or 5 cm internal insulation thickness; (**a**) variable internal gains; (**b**) fixed internal gains.

The variation of temperature and relative humidity on the internal surface were analysed for all walls and ventilation variants, taking into account variable and constant occupancy schedules. Due to the considerable number of diagrams, they were not included in this paper; however, the author included in Figure 2 exemplary diagrams, showing the annual seasonal fluctuations in temperature and relative humidity on the inner surface of the selected variants. The values of maximum and minimum temperature for particular types of wall and ventilation variants and RH values are presented in Table 5 and Table 6, respectively.

Annual seasonal variation in temperature and RH on the interior surface of the walls is evident for all analysed cases. Variant V1 with the lowest intensity of ventilation n = 0.2 h^−1^ shows longer periods of maintaining higher temperature (from mid-March to the end of October) than the remaining variants V2, V3, and V4 (from the beginning of May to the end of September). Obviously, in variant V4, due to the lack of cooling, i.e., stabilisation of the temperature at a certain level (t = 26 °C), in the summer period, the temperature gradually increases and, then, after reaching its maximum at the turn of July and August, it gradually decreases until it reaches its minimum at the level of 20 °C.

With the varying load profile for wall A (perlite board), the lowest recorded temperature value was 17.0 °C (this value was recorded with different ventilation variants V3 and V4 and different insulation thicknesses). The highest value is 39.8 °C for variant V4 with 12 cm of insulation. For wall B (microporous Ca-Si), the lowest value was 16.9 °C, while the highest was 39.9 °C. For walls C (rigid wood fibreboard) and D (flex wood fibreboard), the values were 16.5 °C and 17.0 °C, and 39.3 °C and 39.8 °C, respectively. The highest values of minimum temperature were recorded for ventilation variants V1 and V2, while the lowest for variant V4. Conversely, in the case of maximum values, the opposite applies, where the highest values of maximum temperature on the inner surface were recorded for variant V4 and the lowest for variant V1. There were no significant differences between the obtained temperature values for the different variants of wall system solutions with the same ventilation and insulation variants. A minimal increase in temperature values in the range of 0.05–1 °C can be observed depending on the ventilation variant with increasing insulation thickness. For variants V1 and V2, the average minimum temperature on the inner surface was 19.5 °C and the maximum was 27 °C. On the contrary, for variants V3 and V4, the average minimum temperature was 16.54 °C, whereas the maximum for variant V3 does not differ from variants V1 and V4, and is 39.3 °C. Lower temperature for variant V3 is the result of more intensive ventilation, while higher temperature for variant V4 is the result of the absence of a cooling option for this variant.

In the case of the constant occupancy schedule, the same regularities were observed, but for ventilation variants V3 and V4, the minimum temperature value is on average by 0.3 °C, and at its maximum by 2.6 °C, higher than for the variable occupancy schedule (for all walls), whereas, for variant V1, the minimum temperature value is on average 2.1 °C higher for all walls. Interestingly, in variant V2, there was no difference in the minimum and maximum temperature values between the constant and variable occupancy schedules. Lower values of minimum temperature, below 20 °C for variants V3 and V4, are the result of increased ventilation due to more intensive airing by opening the window.

Regardless of the wall type (A, B, C, D) for a given ventilation variant, the same minimum and maximum RH values were obtained for both variable and constant load profiles. Moreover, the minimum and maximum RH values do not change with increasing insulation thickness. The tests by [42] also showed similar results. Increasing the internal insulation thickness beyond 100 mm did not change the maximum and minimum RH values on the internal surface of the wall.

For the variable load profile, the lowest minimum RH = 17–18% was recorded in variants V4 and V3, which corresponds to a maximum RH = 52–50% in variant V4 and RH = 67–65% in variant V3. The highest minimum value of RH = 37–38% was recorded in variant V1, which corresponds to the maximum value of RH = 73–74%. Conversely, for variant V2, the minimum RH = 22–23% and the maximum RH = 66–67% were obtained. The obtained minimum and maximum RH values can be ranked from highest to lowest in the following order for each ventilation variant V1 > V2 > V3 > V4.

For the constant load profile, the same patterns were observed with higher RH values of min RH = 43–45%, max RH = 81–82% for V1, min RH = 26–27%, max RH = 70–73% for V2, min RH = 68–69%, max RH = 20–21% for V3, and min RH = 17–18%, max RH = 49–51% for V4. Higher RH values in variants V1 prove that higher humidity is maintained in the room due to lower ventilation intensity and, in the case of constant occupancy schedules, additionally, higher humidity gains are related to the constant presence of people. A similar situation occurs with respect to temperature. More intensive ventilation is able to take away the heat gains accumulated in the room, which contributes to a lower temperature value on the internal surface of the wall.

### 4.3. TOW and RHTT Indexes

The external insulation of the base wall in the form of EPS board certainly reduces the risk of condensation inside the wall; however, when installing additional internal insulation, the hygrothermal conditions in the wall should be checked each time. The level of relative humidity in the cross-section at the interface between the base wall and the interior insulation has repeatedly been analysed in great detail in the literature [16,19] due to the increased risk of mould and interstitial condensation in this section. The relative humidity at the interface between the masonry and interior insulation depends, in addition to the effects of WDR, on the relative humidity outside and inside the room transmitted by diffusion and air leakage [39]. In addition, the moisture condition of a wall is very significantly affected by the moisture properties of the various materials of which it is constructed.

In order to avoid the risks associated with the occurrence of moisture, the wall must maintain a balance between dampness and rapid drying. The TOW index defined in Section 3 can be used to assess this situation. The calculated values of TOW and RHTT indexes, on the basis of obtained variation patterns of temperature and relative humidity RH (not included in the text because of their relatively large number—available on request) in the cross-section between the additional layer of internal insulation and the base wall are summarised in Table 7. Analysing individual values of TOW, we can see that only in the case of ventilation variant V1, with minimal air exchange n = 0.2 h^−1^, the probability of wall dampness appears. For the constant occupancy schedules, maximum values of TOW = 8760 h were recorded for each wall A, B, C, and D at 12 cm thickness, and minimum values at 4 or 5 cm thickness, with TOW = 0 for wall A (perlite board) and C (rigid wood fibreboard). In general, wall D (flex wood fibreboard) has the highest susceptibility to dampness followed in decreasing order by walls B, A and C.

In the case of the variable occupancy schedule, the probability of dampness only applies to variants with 12 cm thick insulation. In this case, however, the walls line up in a different order. The highest probability occurs in wall D, followed by B, C and finally A. The difference between walls A and C, however, is minimal, and the TOW for wall C is 112 h higher than for wall A.

At the same time, the probability of mould growth on a wall (RHTT index), as well as the probability of dampness (TOW index), appear only in the case of ventilation variant V1 and increase with the number of hours of dampness of a wall and the thickness of additional insulation. The highest RHTT = 7.9 × 10^9^ was recorded for wall D with a constant occupancy schedule and 12 cm of additional insulation. Compared to the other walls A, B, and C, with the same insulation thickness, which also have a TOW value of 8760 h, the RHTT is 1.46, 1.13, and 1.61 times higher, respectively. In contrast, in the case of variable moisture load profiles, the probability of mould occurrence appears for walls B, C, and D with 12 cm of insulation. The highest value of RHTT = 1.7 × 10^8^, i.e., the highest probability of mould occurrence, was recorded for wall D and the lowest RHTT = 1.8 × 10^4^ for wall C.

The least favourable solution is, therefore, wall D with flex wood fibreboard insulation. Analysing the differences between RH values obtained by individual walls, we see that they are negligible. In the case of small insulation thickness 4–5 cm for all ventilation variants, the highest RH values are characteristic for wall B followed by walls A, D, and C. This order changes with higher insulation thicknesses of 8 and 12 cm, in which case the highest RH value is characteristic for wall D and, then, in appropriate order, for walls B, A, and C. This situation occurs both with variable and constant occupancy schedules of rooms. The lowest RH value, in each case, occurs in wall C, a solution based on rigid wood fibreboard with the highest value of diffusion resistance regardless of insulation thickness. Conversely, wall A (perlite board) at 5 cm thickness has relatively high RH due to its high capillary moisture transport capacity [8], which at lower thickness causes faster saturation, hence the higher RH values in this case and the higher total moisture content (Section 2.1). Both walls D (flex wood fibreboard) and C (Ca-Si) are characterised by low diffusion resistance, hence their higher RH values, especially at higher insulation thicknesses. Similar results were found in [22,52].

In addition, with increasing insulation thickness, seasonal fluctuations of RH decrease, and this is much more evident for variants with lower ventilation intensity (V1 and V2) than for variants V3 and V4.

Of course, the obtained results confirm that as moisture loads are reduced (cases with variable occupancy schedule), the hygrothermal conditions in the cross-section between the insulation and the base wall improve due to the reduced vapour diffusion potential to the outside caused by the reduced vapour pressure in the room. These results are in line with previous findings in [22,29,30].

## 5. Conclusions

This paper presents a study of an exterior wall insulated from the inside using four different capillary active open diffusion interior insulation systems. The primary objective was to assess the hygrothermal behaviour of the different solutions under varying indoor conditions. Based on the analyses, one might draw the following conclusions:

- The profiles of the total moisture content for all analysed walls show the cyclic seasonality of moisture content caused by seasonal shifts in climatic conditions.
- The total moisture content of a wall increases with the increase of insulation thickness. Furthermore, an increase in insulation thickness causes an increase in the RH level inside the internal insulation, which significantly reduces the possibility of the wall drying out into the room and reducing the seasonality of the RH level inside the insulation, which in turn reduces the susceptibility of the wall to damage.
- The obtained values of temperature and RH on the internal surface are mainly influenced by the conditions in a room depending on the heating and ventilating systems and internal heat gains. Lack of proper ventilation with higher moisture gains in the room cause an increase in RH and temperature on the internal surface of the wall.
- Analysis of RHTT and TOW indices has shown that the highest risk of mould growth and interstitial condensation is typical for wall D with flex wood fibreboard. This wall also has the highest profile of relative humidity in the analysed section. On the contrary, the lowest RH values were recorded for wall C with rigid wood fibreboard, which has the highest diffusion resistance.

The analyses carried out in this study for four different internal insulation systems allowed for their hygrothermal assessment under different conditions. Due to the large number of parameters that influence the hygrothermal behaviour of a wall, it is impossible to carry out this assessment without using professional computing tools. Therefore, in order to ensure a safe solution, one should carry out an individual assessment at the design stage. This approach will help to avoid significant damage to walls during building occupancy caused by moisture problems. The results of this study will broaden the design knowledge of internally insulated envelopes, as they help to understand how and under what conditions open diffusion capillary active internal insulation can be safely used.

## Figures and Tables

**Table 1:** Wall assembly configurations used in simulations.

Wall Assemblies (Additional Material Layers)	Thermal Conductivity (W/(m·K))	Heat Capacity (J/(kg·K))	Density (kg/m^3^)	μ-Value (–)	Thickness (m)	U-Value (W/(m^2^·K))
A—						
bonding mortar	0.800	850	1350	16.2	0.05	0.176
perlite board	0.045	850	850	7.0	0.08 0.12	0.158 0.138
mineral plaster	0.800	850	190	25.0		
B—						
adhesive mortar	0.155	850	833	15.0	0.05	0.174
microporous Ca-Si	0.043	850	115	4.1	0.08 0.12	0.156 0.136
adhesive mortar	0.155	850	833	15.0		
lime plaster	0.700	850	1600	7.0		
C—					0.04	0.183
rigid wood fibreboard	0.045	2100	159	10.0	0.08 0.12	0.158 0.138
bonding mortar	0.800	850	1350	16.2		
lime plaster	0.700	850	1600	7.0		
D—					0.04	0.180
flex wood fibreboard	0.041	2100	61	3.0	0.08 0.12	0.150 0.133
gypsum fibreboard	0.300	1200	1153	16.0		

**Table 2:** Variants of estimated air exchange.

Variants	Simulated Scenario
V1	infiltration n = 0.2 h^−1^ with cooling (max. temp. 26 °C)
V2	infiltration n = 0.5 h^−1^ with cooling (max. temp. 26 °C)
V3	infiltration n = 0.5 h^−1^ + ajar window over a period of 1 h at 8 a.m. and 6 p.m. (ACH 4.0 h^−1^) with cooling (max. temp. 26 °C)
V4	infiltration n = 0.5 h^−1^ + ajar window over a period of 1 h at 8 a.m. and 6 p.m. (ACH 4.0 h^−1^ ) without cooling

**Table 3:** Occupancy schedule with internal heat gains.

Occupancy Hours	Weekdays				Weekend			
	Heat Conv.	Heat Radiant	Moisture	CO_2_	Heat Conv.	Heat Radiant	Moisture	CO_2_
	W	W	g/h	g/h	W	W	g/h	g/h
6.00–7.00	80	41	59	36.3	–	–	–	–
7.00–8.00	220	112	168	106.4	–	–	–	–
8.00–10.00	–	–	–	–	80	41	59	36.3
10.00–16.00	–	–	–	–	220	112	168	106.4
18.00–20.00	220	112	168	106.4	220	112	168	106.4
20.00–22.00	160	82	118	72.6	160	82	118	72.6

**Table 4:** Maximum and minimum values for total moisture content.

Wall Assemblies	Total Moisture Content [kg/m^3^]	Ventilation Variants											
		V1			V2			V3			V4		
		Insulation Thickness [cm]											
		4–5	8	12	4–5	8	12	4–5	8	12	4–5	8	12
		constant occupancy schedule											
A	min	1.53	1.69	2.06	1.04	1.27	1.53	0.97	1.16	1.40	0.96	1.15	1.38
	max	2.74	3.19	3.65	2.59	2.97	3.37	2.56	2.97	3.37	2.56	2.97	3.37
B	min	1.20	1.41	1.75	0.89	1.20	1.20	0.82	0.96	1.12	0.82	0.94	1.04
	max	2.74	2.96	3.32	2.43	2.99	2.99	2.40	2.65	2.97	2.40	2.65	2.97
C	min	1.53	1.69	2.06	1.04	1.04	1.27	0.97	1.16	1.40	0.96	1.15	1.38
	max	2.74	3.19	3.65	2.59	2.59	3.37	2.56	2.97	3.37	2.56	2.97	3.37
D	min	1.05	1.41	1.75	0.89	1.20	1.20	0.82	0.96	1.12	0.82	0.94	1.04
	max	2.96	2.96	3.32	2.43	2.99	2.99	2.40	2.65	2.65	2.40	2.65	2.97
		variable occupancy schedule											
A	min	1.77	2.12	2.82	1.12	1.37	1.66	0.97	1.30	1.51	0.98	1.15	1.33
	max	2.87	3.56	4.03	2.61	2.99	3.39	2.56	2.97	3.37	2.56	2.97	3.37
B	min	1.44	1.82	2.47	0.97	1.12	1.33	0.88	1.02	1.12	0.81	0.91	1.12
	max	2.87	3.17	3.55	2.45	2.69	3.01	2.40	2.65	2.97	2.40	2.65	2.97
C	min	1.77	2.12	2.82	1.12	1.37	1.66	0.97	1.30	1.51	0.98	1.15	1.33
	max	2.87	3.56	4.03	2.61	2.99	3.39	2.56	2.97	3.37	2.56	2.97	3.37
D	min	1.21	1.82	2.47	0.97	1.12	1.33	0.88	1.02	1.12	0.81	0.91	1.12
	max	2.80	3.17	3.55	2.45	2.69	3.01	2.40	2.65	2.97	2.40	2.65	2.97

**Table 5:** Maximum and minimum values of temperature on the inner surface of the wall.

Wall Assemblies	Temperature [^o^C]	Ventilation Variants											
		V1			V2			V3			V4		
		Insulation Thickness [cm]											
		4–5	8	12	4–5	8	12	4–5	8	12	4–5	8	12
		constant occupancy schedule											
A	min	21.5	21.6	21.8	19.4	19.5	19.5	17.3	17.3	17.4	17.3	17.3	17.1
	max	26.0	26.0	26.0	26.0	26.0	26.0	26.0	26.0	26.0	41.4	42.0	35.7
B	min	21.4	21.6	21.8	19.4	19.5	19.5	17.2	17.2	17.0	17.2	17.2	17.3
	max	26.0	26.0	26.0	26.0	26.0	27.1	26.0	26.0	27.1	41.6	42.1	42.6
C	min	21.3	21.5	21.7	19.4	19.4	19.5	16.9	17.0	17.6	16.9	17.6	17.6
	max	26.0	26.0	26.0	26.0	26.0	26.0	26.0	26.0	26.0	41.3	41.6	42.0
D	min	21.5	21.7	21.9	19.4	19.5	19.6	17.4	17.4	17.5	17.4	17.4	17.5
	max	26.0	26.0	26.0	26.0	26.0	26.0	26.0	26.0	26.0	41.2	42.0	42.5
		variable occupancy schedule											
A	min	19.5	19.5	19.6	19.4	19.5	19.5	17.0	17.0	17.1	17.0	17.0	17.1
	max	26.0	26.0	26.0	26.0	26.0	26.0	26.0	26.0	27.1	38.8	39.3	39.8
B	min	19.5	19.5	19.6	19.4	19.5	19.5	16.9	16.9	17.0	16.9	16.9	17.0
	max	26.0	26.0	27.1	26.0	26.0	27.1	26.0	27.1	27.1	39.0	39.5	39.9
C	min	19.4	19.5	19.6	19.4	19.4	19.5	16.5	17.3	17.4	16.5	17.3	17.4
	max	26.0	26.0	26.0	26.0	26.0	26.0	26.0	26.0	26.0	38.7	38.9	39.3
D	min	19.5	19.5	19.6	19.4	19.5	19.6	17.0	17.1	17.2	17.0	17.1	17.2
	max	26.0	26.0	26.0	26.0	26.0	26.0	26.0	26.0	26.0	38.6	39.3	39.8

**Table 6:** Maximum and minimum values of RH on the inner surface of the wall.

Wall Assemblies	RH [%]	Ventilation Variants											
		V1			V2			V3			V4		
		Insulation Thickness [cm]											
		4–5	8	12	4–5	8	12	4–5	8	12	4–5	8	12
		constant occupancy schedule											
A	min	45	44	44	27	27	27	21	21	20	20	20	21
	max	81	81	82	71	71	71	69	69	69	51	50	64
B	min	45	45	45	27	27	27	21	21	21	21	20	20
	max	80	80	80	70	70	70	68	68	68	50	50	49
C	min	45	45	45	28	27	27	21	21	21	21	21	21
	max	80	80	80	70	70	70	68	68	68	50	50	49
D	min	44	43	43	26	26	26	20	20	20	20	20	20
	max	82	82	82	71	71	71	69	70	70	51	51	50
		variable occupancy schedule											
A	min	37	37	37	23	23	23	18	18	18	18	18	18
	max	73	73	73	67	67	67	67	66	66	51	50	50
B	min	37	37	37	23	23	23	18	18	18	18	18	18
	max	72	72	72	66	66	66	66	66	66	51	50	50
C	min	38	38	38	23	24	24	18	18	18	18	18	18
	max	72	72	72	66	66	66	66	65	65	51	50	50
D	min	37	37	37	23	22	22	17	17	17	17	17	17
	max	73	74	74	67	67	67	67	67	67	52	51	50

**Table 7:** TOW and RHHT indexes.

Wall Assemblies		Ventilation Variants											
		V1			V2			V3			V4		
		Insulation Thickness [cm]											
		4–5	8	12	4–5	8	12	4–5	8	12	4–5	8	12
constant occupancy schedule													
A	TOW [h]	0	4780	8760	0	0	0	0	0	0	0	0	0
	RHTT [-]	0	3.9 × 10^8^	5.4 × 10^9^	0	0	0	0	0	0	0	0	0
B	TOW [h]	351	5264	8760	0	0	0	0	0	0	0	0	0
	RHTT [-]	3.0 × 10^5^	9.8 × 10^8^	7.0 × 10^9^	0	0	0	0	0	0	0	0	0
C	TOW [h]	0	4425	8760	0	0	0	0	0	0	0	0	0
	RHTT [-]	0	6.2 × 10^8^	4.9× 10^9^	0	0	0	0	0	0	0	0	0
D	TOW [h]	921	6131	8760	0	0	0	0	0	0	0	0	0
	RHTT [-]	3.0 × 10^6^	1.6 × 10^9^	7.9 × 10^9^	0	0	0	0	0	0	0	0	0
variable occupancy schedule													
A	TOW [h]	0	0	0	0	0	0	0	0	0	0	0	0
	RHTT [-]	0	0	0	0	0	0	0	0	0	0	0	0
B	TOW [h]	0	0	835	0	0	0	0	0	0	0	0	0
	RHTT [-]	0	0	2.3 × 10^6^	0	0	0	0	0	0	0	0	0
C	TOW [h]	0	0	112	0	0	0	0	0	0	0	0	0
	RHTT [-]	0	0	1.8 × 10^4^	0	0	0	0	0	0	0	0	0
D	TOW [h]	0	0	1905	0	0	0	0	0	0	0	0	0
	RHTT [-]	0	0	1.7 × 10^8^	0	0	0	0	0	0	0	0	0

## Data Availability

The data presented in this study are available from the author upon reasonable request.
